# Supplementary material for: An open-source framework for large-scale, flexible evaluation of biomedical text mining systems
Source: J Biomed Discov Collab. 2008 Jan 29;3:1. doi: 10.1186/1747-5333-3-1 (PMC2276192; doi:10.1186/1747-5333-3-1)
Supplement: Additional file 1 — Evaluation platform source code. The additional file contains the software evaluation framework discussed in this paper. The most current release can be downloaded from SourceForge.net [1]. See the accompanying README file for instructions on installation and use. Also included in the distribution is a collection of UIMA wrappers for some commonly used BioNLP tools and annotated corpora. [file 1747-5333-3-1-S1.zip › bionlp_uima_repository-1.0.1/docs/api/src-html/edu/uchsc/ccp/util/RunExecutable.html]

```
001    /*
002     *
003     * The Original Code is UCHSC Utilities.
004     *
005     * The Initial Developer of the Original Code is University of Colorado.  
006     * Copyright (C) 2005.  All Rights Reserved.
007     *
008     * UCHSC Utilities was developed by the Center for Computational Pharmacology
009     * (http://compbio.uchsc.edu) at the University of Colorado Health 
010     *  Sciences Center School of Medicine with support from the National 
011     *  Library of Medicine.  
012     *
013     *
014     * Contributor(s):
015     *   William A. Baumgartner <william.baumgartner@uchsc.edu> (Original Author)
016     *   
017     * Created: March 2005
018     * Dependencies:
019     * ToDo: 
020     * Changes:
021     */
022    package edu.uchsc.ccp.util;
023    
024    import java.io.BufferedReader;
025    import java.io.InputStreamReader;
026    
027    /**
028     * This class is a simple utility for calling command line executables.
029     * @author William A. Baumgartner, Jr.
030     */
031    public class RunExecutable {
032    
033        /**
034         * Run a command line executable.
035         * @param command the command line function
036         * @param showOutput display output flag. Keep in mind that sometimes displaying output can be necessary to keep the Java process from moving on before the command line function completes.
037         */
038            public static void run(String command, boolean showOutput) {
039                    try {
040                            Process p = Runtime.getRuntime().exec(command);
041    
042                            if (showOutput) {
043                                    BufferedReader ls_in = new BufferedReader(
044                                                    new InputStreamReader(p.getInputStream()));
045                                    BufferedReader ls_err = new BufferedReader(
046                                                    new InputStreamReader(p.getErrorStream()));
047    
048                                    String ls_str;
049    
050                                    System.out.println("--------- INPUT STREAM ---------");
051                                    while ((ls_str = ls_in.readLine()) != null) {
052                                            System.out.println(ls_str);
053                                    }
054                                    ls_in.close();
055    
056                                    System.out.println("----------- ERROR STREAM ----------");
057                                    while ((ls_str = ls_err.readLine()) != null) {
058                                            System.out.println(ls_str);
059                                    }
060                                    ls_err.close();
061                            }
062    
063                            p.waitFor();
064    
065                    } catch (Exception ioe) {
066                            System.out.println("IOException while running executable...");
067                            ioe.printStackTrace();
068                    }
069    
070            }
071    
072            /**
073             * This method used for testing purposes only.
074         * @param args
075             *            the command line arguments
076             */
077            public static void main(String[] args) {
078                    // TODO code application logic here
079    
080                    //String execStr = "ssh hal
081                    // '/sandata1/medline_files/schema/loadPMIDs.bat'";
082    
083                    try {
084                            Process p = Runtime.getRuntime().exec(args[0]);
085    
086                            BufferedReader ls_in = new BufferedReader(new InputStreamReader(p
087                                            .getInputStream()));
088                            BufferedReader ls_err = new BufferedReader(new InputStreamReader(p
089                                            .getErrorStream()));
090    
091                            String ls_str;
092    
093                            System.out.println("--------- INPUT STREAM ---------");
094                            while ((ls_str = ls_in.readLine()) != null) {
095                                    System.out.println(ls_str);
096                            }
097                            ls_in.close();
098    
099                            System.out.println("----------- ERROR STREAM ----------");
100                            while ((ls_str = ls_err.readLine()) != null) {
101                                    System.out.println(ls_str);
102                            }
103                            ls_err.close();
104    
105                            p.waitFor();
106    
107                    } catch (Exception ioe) {
108                            System.out.println("IOException while running executable...");
109                            ioe.printStackTrace();
110                    }
111            }
112    
113    }
```
